# Cytokinesis-Blocking Micronucleus Assay for Assessing Nuclear Chromatin Integrity Abnormalities in Dog’s Somatic Cells After Exposure to HVAD-Produced Silver Nanoparticles

**DOI:** 10.3390/ijms252312691

**Published:** 2024-11-26

**Authors:** Anna Grzesiakowska-Dul, Marek Jan Kasprowicz, Agnieszka Otwinowska-Mindur, Przemysław Baran, Marta Kuchta-Gładysz

**Affiliations:** 1 Department of Animal Reproduction, Anatomy and Genomics, University of Agriculture in Krakow, Mickiewicza Av. 24/28, 30-059 Kraków, Poland; marta.kuchta-gladysz@urk.edu.pl; 2 Department of Soil Science and Agrophysics, University of Agriculture in Krakow, Mickiewicza Av. 21, 31-120 Kraków, Poland; marek.kasprowicz@urk.edu.pl; 3 Department of Genetics, Animal Breeding and Ethology, University of Agriculture in Krakow, Mickiewicza Av. 24/28, 30-059 Kraków, Poland; agnieszka.otwinowska@urk.edu.pl; 4 Veterinary Clinic “Salamandra”, Nowowiejska Street 3, 30-052 Kraków, Poland

**Keywords:** chromatin integrity, dog, silver nanoparticles, toxicity, micronucleus assay

## Abstract

The widespread use of silver nanoparticles in many industries is increasing every year. Along with this use, there is growing concern about the potential unintentional exposure of human and animal organisms to these nanomaterials. It has been shown that AgNPs have the ability to penetrate organisms and can have harmful effects on cells and organs in the body. In order to reduce the effects of AgNPs on living organisms, newer solutions are being investigated, such as particle stabilization or other methods of synthesizing these particles. The physical synthesis of AgNPs using high-voltage arc discharge (HVAD) may be one of these alternatives. To determine the effect of silver nanoparticles obtained by this method, cytogenetic analysis was performed on domestic dog somatic cells using a cytokinesis-blocking micronucleus assay. In the experiments performed, peripheral blood cells of the domestic dog were exposed in vitro for 3 and 24 h to three tested colloidal silver compounds (unstable AgNP-HVAD, sodium citrate-stabilized silver nanoparticles—AgNP+C, and silver nitrate). The toxicity of these compounds was evaluated at concentrations of 5, 10, and 20 µg/L, and the presence of the following cellular abnormalities was analyzed: micronuclei, nuclear buds, nucleoplasmic bridges, or multinucleated cells. The study showed a significant increase in the number of micronuclei compared to the control sample, as well as the presence of nuclear buds and nucleoplasmic bridges in somatic cells of the domestic dog, confirming the genotoxic nature of the particles. However, there was no cytotoxic effect due to the lower number of multinucleated cells and the absence of apoptotic or necrotic cells in the samples analyzed. Further studies are needed to better understand the mechanisms of toxicity of AgNPs produced by the HVAD method and the extent of their effects on mammalian somatic cells.

## 1. Introduction

Nanomaterials, including silver nanoparticles, are becoming an important part of our daily lives. The production and use of silver nanoparticles, which are used in many everyday products due to their good antibacterial, optical, or electrical properties, are increasing every year. The increase in the global demand for nanomaterials increases their penetration into the environment and thus their potential biohazard to humans and animals [1,2].

The aspect of possible interactions with living organisms and the impact on their genome is a very important element in the use of AgNPs in products or in the development of new methods to obtain or use them. Due to their small size, AgNPs can penetrate cell membranes and interact with cellular elements, which can disrupt their function [3,4]. Silver nanoparticles have been shown to enter the human and animal body through up to four routes—dermal absorption, inhalation, ingestion, or deliberate injection [5]. In addition, these small particles can enter the bloodstream and later be transported to internal organs, where they have the ability to accumulate. In addition, the absorption of silver nanoparticles and their metabolic transformation in soft tissues is an important issue of such uptake [3,6].

The available data show that regardless of the route of entry of AgNPs into the body, they are further transported to soft tissues and organs, leading to their dysfunction and cellular damage. These observations apply to the respiratory system including the lungs, the brain, the liver, the intestines, the kidneys, the blood, the reproductive systems of both females and males, and the nervous system. Damage at the level of the hematopoietic and circulatory systems results in the transmission of these abnormalities throughout the body, thereby weakening the health of such an individual.

Many literature data indicate potential cytotoxic and genotoxic effects of AgNPs on eukaryotic cells, but there are many factors that determine these particle properties, such as shape, particle size, method of synthesis, and stabilization [3,7,8,9]. The adverse effects of nanomaterials on living organisms may have a significant impact on the stability of their genome. Increased genome instability, including the accumulation of DNA damage, may lead to the development of mutations and an increase in tumorigenesis [10]. The toxic effects of silver nanoparticles on eukaryotic cells may result from their induction of reactive oxygen species (ROS), followed by oxidative stress, cell cycle disruption, or interaction with proteins and enzymes in cells, thereby altering the metabolism of mammalian cells [3,7,8,9,11].

The micronucleus assay with cytokinesis blocks is of the greatest interest and applied in studies of the genotoxicity of nanomaterials, biomonitoring, and biological dosimetry. The great advantage of this test is the analysis of numerous abnormalities such as micronuclei, nuclear buds, nucleoplasmic bridges, and multinucleated cells that are identified in cells. The analysis of the cytome by the CBMN assay makes it possible to distinguish biomarkers of DNA damage from markers of cytotoxicity [12]. Indicators of DNA damage in lymphocytes include binuclear cells with a micronucleus, binuclear cells with a nucleoplasmic bridge (NPB), and BN cells with a nuclear bud (NBUD). Mononuclear cells with a micronucleus or bud are also such markers. On the other hand, apoptotic and necrotic cells and the ratio of mononuclear cells:binuclear cells:multinuclear cells are considered to be biomarkers of cytotoxicity [12]. The analysis of genotoxicity and cytotoxicity of silver nanoparticles by the CBMN assay has been performed by, among others, AshaRani et al. [8], Kawata et al. [13], Li et al. [14], Tavares et al. [15], Bastos et al. [16], Wang et al. [17], Heshmati et al. [18], and Wang et al. [19].

A continuing major problem with nanomaterials, particularly silver nanoparticles, is the lack of specific information on their level of toxicity. Due to the possibility of many modifications at the manufacturing stage, realistically, particles with slightly different properties are obtained each time. These changes in the physical and chemical properties of AgNPs, however small, may lead to different effects in biological systems. For this reason, we are always dependent on general information about their effects on living organisms. This issue is also relevant to our health, given the wide availability of products containing silver nanoparticles on the consumer market.

Therefore, in our study, we sought to determine the toxicity of silver nanoparticles on nuclear chromatin in animal somatic cells. An innovative aspect of our research was the use of silver nanoparticles obtained by the physical method of HVAD—high-voltage arc discharge. This method produces colloidal solutions of AgNPs quickly and cheaply, with no residual solution of the chemicals or reducing agents used in the reaction. This approach to particle synthesis theoretically allows fewer toxic particles to be obtained. An important aspect of the study was the use of two solutions, one with lower stability and an AgNP solution additionally stabilized with sodium tricitrate, which was also a challenge in our study. The AgNP toxicity data available in the literature are often based on the use of high particle concentrations, whereas in our study we assumed that low concentrations of AgNPs would show toxic effects, so we used concentrations in the range of 5, 10, and 20 µg/L. In addition, an important aspect of the studies conducted was the evaluation of the effects of AgNPs on the cells of the domestic dog, a human companion organism. This aspect is an additional innovative factor as it shows how accidental intoxication with low doses of AgNPs can also affect human cells.

The aim of the study was to determine the extent of nuclear chromatin damage in canine lymphocytes following exposure to colloidal silver nanoparticles produced by the HVAD physical method. Cytome analysis, including changes in the integrity of nuclear chromatin in the cells, was conducted as a function of the following experimental factors: a colloidal silver solution, its concentration, and the exposure time of cells in in vitro conditions using the cytokinesis-blocking micronucleus assay.

## 2. Results

**Figure 1:**
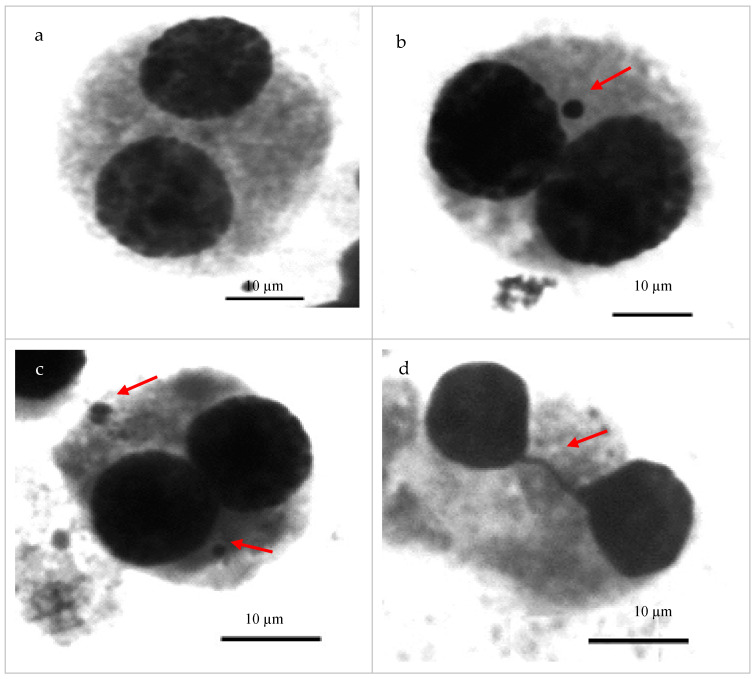

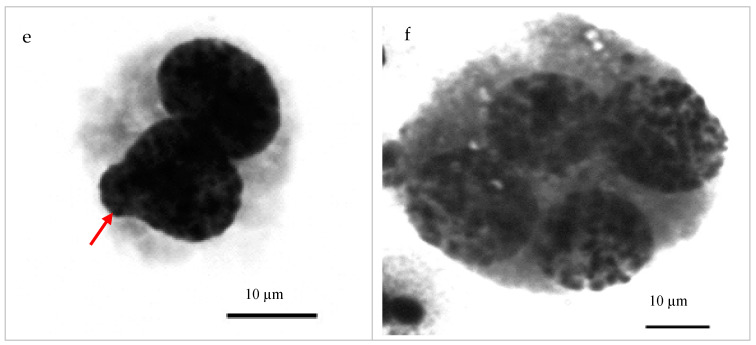
Cells analyzed by CBMN assay in domestic dog samples: (**a**) binucleated cell; (**b**) binucleated cell with one micronucleus; (**c**) binucleated cell with two micronuclei; (**d**) binucleated cell with nucleoplasmic bridge; (**e**) binucleated cell with nuclear bud; (**f**) multinucleated cell. Giemsa staining, 100× objective magnification, 10 µm scale. Arrows indicate forms of chromosomal instability.

The following cell types and abnormalities were identified and the frequency of their occurrence was analyzed in the cytokinesis inhibition micronucleus assay for the canine lymphocyte experiment: BNC—binucleated cell; BNC+1MN—binucleated cell with one micronucleus; BNC+2MN—binucleated cell with two micronuclei; NPB—nucleoplasmic bridge binucleated cell; NBUD—nuclear bud binucleated cell; MNC—multinucleated cell (Figure 1).

In domestic dog lymphocytes (Figure 1b,c), the occurrence of micronuclei (1 or 2) in binucleated cells obtained by the CBMN assay was analyzed. The effect of silver colloidal solutions with respect to the control group was determined as the frequency of BNCs with one or two micronuclei per average number of BNC+1MN and BNC+2MN, as shown in Table 1. In the control group, BNCs with one micronucleus were found at the level of 11.00 ± 4.42.

There were no significant differences between the tested concentrations of 5, 10, and 20 µg/L in the analysis of the effect of the dose of the tested silver solutions on the number of micronuclei observed in the binucleated cells of the domestic dog. The incidence of BNC+1MN was found at an average level of 21.85–24.70 BNC+1MN. Lymphocytes exposed to AgNP-HVAD at concentrations of 5 and 10 µg/L showed an average presence of 22 BNC+1MN, while the highest concentration tested showed a higher damage inducibility of 24.40 BNC+1MN. Also, with silver nitrate, an increase in the number of induced micronuclei was observed after treatment with 20 µg/L (see Table 1 for details). The incidence of BNC+1MN in dog cells after exposure to the tested concentrations of silver compounds was significantly higher than the average number of lesions in the control group for each variant tested. Cells with two micronuclei were rarely identified in dog lymphocytes, with an average of 0.10–0.80 BNC+2MN observed. A higher frequency of this damage was observed only at a concentration of 10 µg/L AgNP-HVAD with 1.30 ± 1.49, and at 5 µg/L AgNO_3_ with 1.10 ± 1.97 BNC+1MN.

There was no effect of this factor on the level of induced micronuclei in binucleated cells of domestic dog lymphocytes in further analysis related to the time of cell exposure to silver compounds and changes in nuclear chromatin stability. Higher values of the average BNC+1MN were observed after 3 h of exposure (24.63–25.17) than after 24 h (21.57–23.33) of exposure of the cells (see Table 1 for details). The number of BNC+2MN was in the range of 0.20–0.77 for domestic dog lymphocytes. Slightly higher values were also observed for this parameter after short-term exposure. The interaction of all analyzed factors, i.e., silver solution, dose, and exposure time, did not affect the average number of BNC+1MN. The observed damage was at an average level of 19.10–26.60 BNC+1MN. The highest number of induced MN was found after short-term exposure at the lowest concentration tested for both silver nanoparticle solutions (AgNP-HVAD and AgNP+C) (Table 1).

As part of the CBMN assay, two other types of cellular abnormalities resulting from changes in nuclear chromatin integrity were also analyzed. The occurrence of nucleoplasmic bridges and nuclear buds (Figure 1d,e) in binucleated cells was evaluated and determined by the parameters of average number of NPBs/sample and average number of NBUDs/sample, respectively. For control samples, the incidence of these abnormalities in domestic dog lymphocytes was determined to be 0.80 ± 1.13 NPBs/sample and 2.10 ± 2.47 NBUDs/sample.

As with the incidence of micronucleus number, the analysis of NPB and NBUD frequencies was performed to evaluate the effect of test compound dose, cell exposure time, and the effect of different experimental variants on the incidence of these abnormalities in domestic dog lymphocytes. The detailed results of these analyses for each parameter are presented in Table 2 and Table 3, respectively.

The frequency of nucleoplasmic bridges in lymphocytes of domestic dogs was not affected by the dose (5/10/20 µg/L) of colloidal silver solutions tested. The average level of damage after the exposure to the silver solutions and their doses was between 0.45 and 1.45 NPBs. For silver nanoparticle solutions (AgNP-HVAD and AgNP+C), the highest frequency of NPBs in cells was induced by a concentration of 10 µg/L (Table 2).

The analysis of the duration of the exposure of domestic dog lymphocytes to the tested silver compounds showed that the average number of NPBs/sample was higher after 3 h of exposure (1.20–2.00 NPBs/sample) than after 24 h of exposure (0.33–0.63 NPBs/sample), regardless of the silver solution used. There was a significant effect of exposure time on the efficacy of the silver solutions tested. The observed differences in the values of the average NPB number/sample between the exposure times of 3 h and 24 h for each silver solution were highly significant (*p* < 0.01; Table 2).

The toxicity evaluation of the different experimental variants revealed that the average number of NPBs/sample varied from 0.02 to 2.30 NPBs/sample in each experimental group. In cells treated with AgNP-HVAD solution at a concentration of 20 µg/L for 24 h of exposure, no nucleoplasmic bridges were observed. The analysis showed a significant effect of the solution—its dose and duration of action—only in two cases: the differences in the average number of NPBs/sample between 3 h and 24 h of exposure time for AgNP-HVAD 20 µg/L and between 3 h and 24 h for AgNO_3_ 5 µg/L were highly significant (*p* < 0.01). There was no great significance between the effects of the two durations on domestic dog cells for the other dose and silver solution variants (Table 2).

In evaluating the effect of silver compound dose on the level of chromatin aberrations, nuclear buds were observed in binucleated cells in the range of 0.40–2.40 NBUDs/sample. The analysis showed a highly significant (*p* < 0.01) effect of the dose of the compound on the level of the observed changes for the two silver solutions tested, between the doses of 5 µg/L and 20 µg/L for AgNP+C and between 5 µg/L and 20 µg/L for AgNO_3_. For the other silver solution and doses, the differences were not significant (Table 3).

On the other hand, 1.17–3.00 NBUDs/sample were identified in the canine cells after 3 h compared to 0.67–1.30 NBUDs/sample after 24 h of exposure when analyzing cell exposure time to each colloidal solution. For each silver solution tested, a higher NBUD frequency was observed after a shorter exposure time. A highly significant effect (*p* < 0.01) of exposure time was observed only for AgNP-HVAD effects between 3 h and 24 h when evaluating the occurrence of nuclear buds in cells. There were no highly significant differences in the average number of NBUDs/sample observed between the exposure times for AgNP+C and AgNO_3_.

The occurrence of binucleated cells with nuclear buds in domestic dog lymphocytes at the level of 0.10–3.60 NBUDs/sample was determined in the analysis of individual experimental variants. There was a significant dependence of the number of induced NBUDs on the silver solution dose and time of action in three cases. Highly significant differences in the average number of NBUDs/sample were found between 3 h and 24 h of exposure for AgNP-HVAD 10 µg/L and AgNP-HVAD 20 µg/L and AgNP+C 20 µg/L (*p* < 0.01). The other experimental variants did not show highly significant differences (Table 3).

Multinucleated cells—multiNCs (MNCs)—present in the samples were noted as part of the CBMN assay (Figure 1f). Their presence is associated with the disruption of cell division. This is associated with changes in chromatin integrity. The average number of multinucleated cells in blood cells was also determined at different stages of analysis of the effect of colloidal silver solutions on nuclear chromatin stability. Table 4 presents data on the effects of the tested silver compounds on this factor.

According to the analysis, a very high value of the average number of MNCs/sample was found in the control group (37.90 ± 32.85 MNCs/sample). The dose analysis of the tested silver colloidal solutions induced the appearance of multinucleated cells at the level of 1.50 to 18.33 MNCs/sample. The highest values of the average number of MNCs were observed at a concentration of 5 µg/L for each silver solution, i.e., AgNP-HVAD, AgNP+C, and AgNO_3_ for the other doses. The analysis showed that the level of induced multiNCs in peripheral blood cells was not affected by the dose of each silver solution. Mean MNCs/sample were not highly significantly different between the doses of silver compounds (Table 4).

On the other hand, the number of MNCs varied from 1.87 to 12.73 MNCs/sample when analyzing the exposure time of peripheral blood cells to silver solutions. For each silver solution, higher values of the parameter were observed after 3 h of exposure. The analysis of the exposure time did not show its effect on the level of induced MNCs in dog lymphocytes. The differences observed for each silver solution between the exposures of 3 h and 24 h were not highly significant (Table 4).

The analysis of the detailed experimental variants showed no effect of the dose dependence of the silver solution and the exposure time. The average number of MNCs/sample between the experimental variants, i.e., between silver colloidal solution/dose/exposure time, was not highly significantly different (Table 4).

The identification of apoptotic cells was the final aspect of the cellular abnormalities analyzed by the CBMN assay on dog lymphocytes after exposure to the colloidal silver solutions tested. Single apoptotic cells were found in the analyzed material (Table 5). Apoptotic cells were present in the range of 0 to 0.50 APCs/sample. There was no effect of solution, exposure time, dose, or the correlation of these factors on the increase in their number in the tested samples for this type of damage.

Based on the data in Table 5, there is a greater predisposition of AgNP-HVAD activity to induce cell apoptosis using the example of domestic dog lymphocytes.

## 3. Discussion

As the production of AgNPs and their use in various industries increases each year, so does the amount of AgNPs released into the environment. Humans, animals, and plants can be exposed to the non-targeted effects of AgNPs through their presence in water, soil, and air. The use of consumer products may result in additional contact/exposure to silver nanoparticles for humans and animals. In assessing the potential exposure of humans and animals to AgNPs, the size and shape of the particles, as well as their concentration and duration of action on biological material, should be considered [20,21,22].

The CBMN variant micronucleus test is one of the methods for the analysis of chromosomal instability. This method is used to determine the genotoxic potential of chemicals and exogenous agents, as well as to monitor chromosome damage in various cell types. The CBMN test is used in vitro to assess the potential toxicity of newly synthesized chemicals [23].

In the present study, the CBMN variant of the micronucleus assay was used to analyze chromatin damage in canine peripheral blood lymphocytes because of the potential to identify other karyokinetic and cytoplasmic damage. The study specified binucleated cells with one or occasionally two micronuclei. In the case of domestic dog cells, the genotoxicity of silver solutions was at a similar level, characterized by the presence of MN in the range of 23.10 to 23.50 BNC+1MN/sample. This was more than twice as high as in the control sample. A significant increase in the frequency of MN in cells after AgNP treatment was also observed by Li et al. [14] in their study. Kawata et al. [13] found significant cytotoxicity after the exposure of HepG2 cells to AgNPs at a level of 47.9% MN/1000 BNC compared to the control group (2.1% MN/1000 BNC) and AgNO_3_ (2.6% MN/1000 BNC) in their study.

In the present study, there was also no significant effect of the dose of silver nanoparticle on the level of micronuclei induced. For AgNP-HVAD, a higher level of damage was observed after a dose of 20 µg/L. The genotoxicity of silver nitrate increased in a dose-dependent manner. AshaRani et al. [8] found the highest level of induced micronuclei after exposure to a dose of 100 µg/L AgNPs, which was approximately 50% MN/BNC, followed by a decrease to 8% MN/BNC at 200 µg/L. A concentration-dependent increase in micronuclei induction in HepG2 cells was demonstrated by Wang et al. [17] after exceeding a concentration of 50 µg/L AgNPs. In the case of the second cell line analyzed by this team, A549, an increase in MN frequency was observed after treatment with 50 and 100 µg/L AgNPs. Micronuclei induction rates were 1.9 and 1.7 times higher than the control, respectively [17]. For both types of silver nanoparticles tested, 20 nm AgNPs and 20 nm PVP-AgNPs, in the concentration range of 80–160 µg/L and 20–160 µg/L, respectively, a statistically significant dose-dependent increase in the number of micronuclei of AgNPs was shown by Wang et al. [19].

In the present studies, there was no time-dependent increase in the number of micronuclei in domestic dog peripheral blood cells. In domestic dog lymphocytes, a higher genotoxicity of the tested solutions was observed after a shorter exposure time of 3 h. There is little information in the available literature about the effect of the exposure time of cells to silver nanoparticles and the increase in the frequency of induced micronuclei in the CBMN assay. Only Tavares et al. [15] in human peripheral blood cells showed that each of the tested doses of AgNPs (10, 25, and 50 µg/L) induced a significant increase in the number of MN. However, with increasing duration of nanosilver exposure, the level of DNA damage gradually decreased. This indicates that at low doses, the genetic toxicity of AgNPs to cells may depend on the cell repair system.

Another indicator of DNA damage in the CBMN assay is the presence of nucleoplasmic bridges in binucleated cells. In the present study, there was a slight increase in the number of NPBs in the domestic dog cells after exposure to AgNP-HVAD. For this type of abnormality, there was no dose effect of the colloidal silver solutions tested. However, time-dependent genotoxicity was confirmed in canine lymphocytes, with a significant decrease in the number of NPBs at 24 h compared to 3 h of exposure observed for each solution. The analysis of nucleoplasmic bridges was reported by Wang et al. [17], who found that AgNPs caused a statistically significant and dose-dependent increase in NPBs at a concentration range of 50–200 µg/L. In addition, they observed that exposure to AgNPs at a concentration of 200 µg/L induced a stronger response in HepG2 cells compared to the A549 cell line, with an 8-fold increase in NPB induction compared to the control group. Thus, they concluded that the dose-dependent increase in NPB frequency was probably due to DNA strand breaks induced by AgNPs. In contrast, in a study by Wang et al. [19], the authors observed no difference in the number of nucleoplasmic bridges after the administration of two types of nanosilver (20 nm AgNPs; 20 nm PVP-AgNPs) at all dose levels tested.

The third CBMN indicator of DNA damage analyzed in this study was the nuclear bud present in a binucleated cell. A relatively high level of NBUDs was observed in the domestic dog control samples (2.10 NBUDs/sample). The exposure of peripheral blood to silver colloidal solutions did not induce this type of damage, and the average number of NBUDs was actually reduced in most samples compared to the control. Only the exposure of canine cells to AgNP-HVAD resulted in similar NBUD levels (2.13 ± 1.66 NBUDs/sample). In addition, a dose-dependent decrease in the number of NBUDs was observed for canine lymphocytes treated with AgNP+C and AgNO_3_ solutions, with significant differences between 5 and 20 µg/L. For the tested colloidal solutions of silver nanoparticles, the observed level of NBUDs was time-dependent. AgNP-HVAD showed a stronger induction at 3 h than at 24 h in domestic dog cells. Similar trends were observed for the other experimental groups. Numerous NBUDs in A549 and HepG2 cells after treatment with AgNPs were observed by Wang et al. [17]. They found a dose-dependent increase in the number of NBUDs compared to the control group. In their study, they also observed that 24 h of exposure to AgNPs resulted in higher NBUD induction in HepG2 cells in the concentration range of 12.5 to 100 µg/L, but a lower index value at the highest concentration of 200 µg/L compared to A549 cells. The highest induction of NBUDs was 11- and 16-fold higher in HepG2 and A549 cells, respectively, compared to the control group [17]. Bastos et al. [16] showed that the number of NBUDs tended to increase after exposure to AgNPs at both application times (24 h and 48 h) and concentrations (10 and 40 µg/L). In contrast, in groups treated with 20 nm AgNPs at 160 µg/L and 20 nm PVP-AgNPs at doses of 20–160 µg/L, Wang et al. [19] observed an increased number of nuclear buds in HepG2 cells.

The presence of biomarkers of cytotoxicity in the analyzed cells was also noted in this study. The analysis of peripheral blood lymphocytes from the domestic dog showed almost no apoptotic or necrotic cells, and thus the cytotoxic nature of AgNP-HVAD, AgNP+C, or AgNO_3_ was not confirmed. On the other hand, there was a significantly lower level of multinucleated cells in the domestic dog experimental groups analyzed than in the control samples. A decrease in the number of multinucleated cells after exposure to AgNPs was also observed by Bastos et al. [16]. In contrast, AshaRani et al. [8] observed relatively few apoptotic and necrotic cells during CBMN analysis. The authors also found no nuclear fragmentation in their analysis.

A challenge of the studies conducted was the inability to explicitly compare the results obtained with other available literature data. These limitations are due to the wide variety of particles in terms of production method, shape, size, concentration, aggregation, sheathing, particle stabilization, duration of action, and material/model organism. Therefore, our results and the literature data can only be compared regarding some aspects of general trends of AgNP action on living organisms. Also, such limitations make it very difficult to determine whether a particular type of particle is a toxic material or not.

## 4. Materials and Methods

### 4.1. Animal

The material for the study consisted of whole peripheral blood collected from the *vena cephalica antebrachi*. Whole peripheral blood was collected from the domestic dog (*Canis familiaris*). The experimental group consisted of 10 individuals. Approximately 2 mL of whole blood was collected from each individual during the veterinary examination in sterile Vacuumed tubes (FL Medical, Torreglia, Italy) contained lithium heparin. Blood samples from domestic dogs were collected at the Salamandra Veterinary Clinic in Krakow by a veterinarian and with the consent of the animals’ owners. The dogs were aged between 3 and 6 years.

### 4.2. Silver Nanoparticles Characteristic

Silver nanoparticles were produced by high-voltage discharge in an electric arc. The production methods and properties of nanoparticles are described in detail in Kasprowicz et al. [24,25]. In a reactor about 1 × 10^−5^ m^3^ in capacity, two electrodes of 10 mm and 5 mm in diameter, made of 99.9% pure silver, were placed, immersed in double-distilled water (AgNP-HVAD) (conductivity − (6−10)*10^−6^ Si − electric conductivity was measured using OK-102/1 conductivity meter Radelkist, Budapest, Hungary) or 3.3 µM water solutions of trisodium citrate dihydrate (TSC; AgNP+C), (pure, POCH, Gliwice, Poland). The voltage between the electrodes was 20 kV. The distance between the electrodes was 2.5 × 10^−4^ m. Using the dynamic light scattering technique (Nano ZS Zetasizer Malvern Instruments Ltd., Worcestershire, UK), the size of the nanoparticles in water was determined to be about 22 nm (AgNP-HVAD) and about 38 nm in TSC (AgNP+C). In order to confirm the nanoparticles sizes, the TEM images (JEOL JEM 100SX transmission electron microscope JEOL, Tokyo, Japan) were analyzed using ImageJ 1.46r computer program (National Institutes of Health, Bethesda, MD, USA). The silver nanoparticles had a spherical shape and diameter of approximately 18 nm (water—AgNP-HVAD and TSC solution—AgNP+C). The zeta potential in colloids was estimated to be −22 mV for water (AgNP-HVAD) and −19 mV for TSC (AgNP+C) using the DLS technique (Nano ZS Zetasizer Malvern Instruments Ltd., Worcestershire, UK). The concentration of silver nanoparticles in colloids was measured by AAS spectrometer (SOLAAR-M6 Thermo Jarrell Ash Co., Midland, TX, USA). UV–Vis spectra were obtained using Specord M40 transmission spectrophotometer (Carl Zeiss Jena, Düsseldorf, Germany) with a computer data acquisition system (Medson, Paczkowo, Poland). UV–Vis spectra had a maximum at 396 nm for water (AgNP-HVAD) and 394 nm for TSC (AgNP+C).

### 4.3. Cells Exposition to Silver Nanoparticles

The experiment was performed on the control sample (cells not exposed to the tested agents) as well as on the cells exposed to the tested silver solutions. The solutions analyzed were as follows: unstable silver nanoparticles in distilled water (AgNP-HVAD), silver nanoparticles in distilled water stabilized with sodium tricitrate solution (AgNP+C), and silver nitrate as an indicator of toxicity at three concentrations (5, 10, and 20 µg/L) during 3 and 24 h of exposure.

### 4.4. Cytokinesis-Blocking Micronucleus Assay

The micronucleus assay was performed according to the method described by Słonina and Gasińska [26]. A fraction of lymphocytes isolated on a Histopaque-1077 (Sigma Aldrich, Poznan, Poland) was used for the assay. After 44 h of lymphocyte culture, cytochalasin B at a concentration of 5 µg/L was added to each cell culture to block cytokinesis. This protocol allows cells that have completed one nuclear division to be recognized as binucleated cells (BNCs) and micronuclei to be scored only in these BNCs [27]. After 72 h of culture, the experimental material was fixed in Carnoy’s solution (3:1 mixture of methanol and acetic acid, POCH, Gliwice, Poland) and then the slides were stained with Giemsa in Sorensen’s buffer. For each experimental sample/individual, 500 binucleated cells (BNCs) were scored according to Fenech criteria [28], where the number of binucleated cells with one micronucleus (BNC+1MN), two micronuclei (BNC+2MN), and other abnormalities such as nucleoplasmic bridges (NPBs), nuclear buds (NBUDs), apoptotic cells (APCs), or multinucleated cells (MNCs) were evaluated.

Microscopic analysis and photographic documentation were performed using a Zeiss Imager A2 epifluorescence microscope coupled with a Zeiss AxioCam MRc5 digital camera (Carl Zeiss, Düsseldorf, Germany) and NIS-Elements image analysis software ver. F2.31 (Nikon, Tokio, Japan).

### 4.5. Statistical Analysis

All calculations were performed with the SAS statistical package (SAS, Cary, NC, USA) [29]. Due to the lack of a normal distribution of the analyzed characteristics, non-parametric tests were performed. In the experiment, the following factors were tested using the Kruskal–Wallis test: the type of solution, the concentration of the solution, and the exposure time. The influence of these factors on the number of binucleated cells with micronuclei, nuclear buds, nucleoplasmic bridges, multinucleated cells, and apoptotic cells was analyzed. For this purpose, the mean values of measurements taken from 10 individuals per species were used. The non-parametric Dwass, Steel, Critchlow-Fligner test was used for multiple comparisons. In this study, all *p*-values less than 0.01 were considered highly statistically significant.

## 5. Conclusions

The types of damage observed in peripheral blood lymphocytes by the CBMN assay confirm the genotoxic nature of the colloidal silver solutions tested, mainly silver nanoparticles. At the same time, the low value of index multinucleated cells and the absence of apoptotic and necrotic cells observed do not indicate the cytotoxic nature of AgNP-HVAD, AgNP+C, or AgNO_3_ towards domestic dog lymphocytes.

The innovative colloidal solutions of silver nanoparticles obtained by physical methods used by us show genotoxic effects in vitro. Studies confirm that even small concentrations of particles, nanomaterials in the range of 5 to 20 µg/L, can cause damage to nuclear chromatin in somatic cells, and consequently have a destabilizing effect on the genome of animals and humans. This is a very important aspect of the use of silver nanoparticles and unintentional intoxication with them. Our studies, despite the intentional effect of low concentrations of AgNPs, show that such cell damage is possible. To obtain more precise data, it seems necessary to perform in vivo studies on an animal model of intoxication with low doses of AgNPs.

## Figures and Tables

**Table 1:** The average number of BNC+1MN in the dog’s blood cells as a function of the effect of a given dose of silver solution at a given time of exposure.

Solutions	Time [h]	Concentration µg/L			Mean Values for the Solution
		5	10	20	
AgNP-HVAD	3	26.10 ± 4.98	24.70 ± 9.18	23.10 ± 7.28	24.63 ± 7.20
	24	19.10 ± 6.06	19.90 ± 4.53	25.70 ± 5.35	21.57 ± 5.97
	mean	22.60 ± 6.48	22.30 ± 7.46	24.40 ± 6.36	-
AgNP+C	3	26.60 ± 7.73	26.60 ± 7.65	22.30 ± 10.03	25.17 ± 8.50
	24	20.40 ± 4.37	20.40 ± 3.72	24.50 ± 3.06	23.33 ± 4.12
	mean	23.50 ± 6.89	23.50 ± 6.66	23.40 ± 7.31	-
AgNO_3_	3	23.70 ± 7.04	26.20 ± 5.43	25.00 ± 7.15	24.97 ± 6.44
	24	20.00 ± 4.71	21.70 ± 6.65	24.40 ± 4.99	22.03 ± 5.63
	mean	21.85 ± 6.13	23.95 ± 6.34	24.70 ± 6.01	-
control		11.00 ± 4.42			

Mean ± standard deviation expressed in number of BNC+1MN/sample.

**Table 2:** The average number of NPBs in the dog’s blood cells as a function of the effect of a given dose of silver solution at a given time of exposure.

Solutions	Time [h]	Concentration µg/L			Mean Values for the Solution
		5	10	20	
AgNP-HVAD	3	1.70 ± 1.95	2.00 ± 1.05	2.30 ± 2.31 ^d^	2.00 ± 1.80 ^a^
	24	1.00 ± 0.94	0.90 ± 0.74	0.00 ± 0.00 ^d^	0.63 ± 0.81 ^a^
	mean	1.35 ± 1.53	1.45 ± 1.05	1.15 ± 1.98	-
AgNP+C	3	1.40 ± 1.26	1.60 ± 1.43	0.60 ± 0.97	1.20 ± 1.21 ^b^
	24	0.40 ± 0.70	0.40 ± 0.52	0.30 ± 0.67	0.37 ± 0.61 ^b^
	mean	0.90 ± 1.12	1.00 ± 1.21	0.45 ± 0.83	-
AgNO_3_	3	2.00 ± 1.63 ^e^	1.40 ± 2.06	0.50 ± 0.85	1.30 ± 1.66 ^c^
	24	0.30 ± 0.48 ^e^	0.20 ± 0.42	0.50 ± 1.27	0.33 ± 0.80 ^c^
	mean	1.03 ± 1.35	0.80 ± 1.58	0.50 ± 1.05	-
control		0.80 ± 1.13			

Mean ± standard deviation expressed in number of NPBs/sample; abcde—values marked with the same letters within columns are highly significantly different at *p* < 0.01.

**Table 3:** The average number of NBUDs in the dog’s blood cells as a function of the effect of a given dose of silver solution at a given time of exposure.

Solution	Time [h]	Concentration µg/L			Mean Values for the Solution
		5	10	20	
AgNP-HVAD	3	2.60 ± 1.95	3.60 ± 1.26 ^e^	2.80 ± 1.40 ^f^	3.00 ± 1.57 ^a^
	24	1.80 ± 1.62	1.10 ± 0.87 ^e^	0.90 ± 1.10 ^f^	1.27 ± 1.26 ^a^
	mean	2.20 ± 1.79	2.35 ± 1.66	1.85 ± 1.56	-
AgNP+C	3	2.70 ± 1.64	1.60 ± 1.62	1.60 ± 0.97 ^d^	1.97 ± 1.50
	24	2.10 ± 1.60	1.50 ± 1.18	0.30 ± 0.67 ^d^	1.30 ± 1.39
	mean	2.40 ± 1.60 ^b^	1.55 ± 1.39	0.95 ± 1.05 ^b^	-
AgNO_3_	3	2.10 ± 1.20	1.30 ± 1.77	0.10 ± 0.32	1.17 ± 1.46
	24	0.70 ± 0.95	0.60 ± 1.07	0.70 ± 1.64	0.67 ± 1.21
	mean	1.63 ± 1.75 ^c^	0.95 ± 1.47	0.40 ± 1.19 ^c^	-
control		2.10 ± 2.47			

Mean ± standard deviation expressed in number of NBUDs/sample; abcdef—values marked with the same letters within columns or rows are highly significantly different at *p* < 0.01.

**Table 4:** The average number of MNCs in the dog’s blood cells as a function of the effect of a given dose of silver solution at a given time of exposure.

Solutions	Time [h]	Concentration µg/L			Mean Values for the Solution
		5	10	20	
AgNP-HVAD	3	19.90 ± 21.31	11.10 ± 14.77	7.20 ± 9.79	12.73 ± 16.36
	24	6.10 ± 6.89	7.60 ± 8.87	1.40 ± 2.46	5.03 ± 6.94
	mean	13.00 ± 21.31	9.35 ± 11.99	4.30 ± 7.56	-
AgNP+C	3	14.40 ± 18.57	8.30 ± 11.29	5.10 ± 5.15	9.27 ± 13.05
	24	6.50 ± 5.90	2.80 ± 3.79	1.10 ± 2.23	3.47 ± 4.70
	mean	10.45 ± 14.01	5.55 ± 8.67	3.10 ± 4.38	-
AgNO_3_	3	14.20 ± 16.88	2.90 ± 4.84	1.90 ± 3.21	6.33 ± 11.45
	24	2.90 ± 4.43	0.10 ± 0.32	2.60 ± 4.97	1.87 ± 3.93
	mean	18.33 ± 25.48	1.50 ± 3.63	2.25 ± 4.09	-
control		37.90 ± 32.85			

Mean ± standard deviation expressed in number of MNCs/sample.

**Table 5:** The average number of APCs in the dog’s blood cells as a function of the effect of a given dose of silver solution at a given time of exposure.

Solutions	Time [h]	Concentration µg/L			Mean Values for the Solution
		5	10	20	
AgNP-HVAD	3	0 ± 0	0.20 ± 0.42	0.10 ± 0.32	0.10 ± 0.24
	24	0.50 ± 1.58	0.10 ± 0.32	0 ± 0	0.20 ± 0.63
	mean	-	-	-	-
AgNP+C	3	0 ± 0	0.20 ± 0.42	0.20 ± 0.63	0.13 ± 0.35
	24	0 ± 0	0 ± 0	0 ± 0	0 ± 0
	mean	-	-	-	-
AgNO_3_	3	0 ± 0	0.20 ± 0.63	0 ± 0	0.07 ± 0.21
	24	0 ± 0	0 ± 0	0.10 ± 0.31	0.03 ± 0.10
	mean	-	-	-	-
control		0 ± 0			

mean ± standard deviation expressed in number of APCs/sample.

## Data Availability

None of the data were deposited in an official repository. The original contributions presented in the study are included in the articles.
